# Primary care contacts, continuity, identification of palliative care needs, and hospital use: a population-based cohort study in people dying with dementia

**DOI:** 10.3399/BJGP.2021.0715

**Published:** 2022-07-12

**Authors:** Javiera Leniz, Martin Gulliford, Irene J Higginson, Sabrina Bajwah, Deokhee Yi, Wei Gao, Katherine E Sleeman

**Affiliations:** NIHR clinician scientist and honorary consultant in palliative medicine, Cicely Saunders Institute for Palliative Care, Policy & Rehabilitation, Florence Nightingale Faculty of Nursing, Midwifery & Palliative Care, King’s College London, London.; Department of Population Health Sciences, Faculty of Life Science & Medicine, King’s College London, London.; NIHR clinician scientist and honorary consultant in palliative medicine, Cicely Saunders Institute for Palliative Care, Policy & Rehabilitation, Florence Nightingale Faculty of Nursing, Midwifery & Palliative Care, King’s College London, London.; NIHR clinician scientist and honorary consultant in palliative medicine, Cicely Saunders Institute for Palliative Care, Policy & Rehabilitation, Florence Nightingale Faculty of Nursing, Midwifery & Palliative Care, King’s College London, London.; NIHR clinician scientist and honorary consultant in palliative medicine, Cicely Saunders Institute for Palliative Care, Policy & Rehabilitation, Florence Nightingale Faculty of Nursing, Midwifery & Palliative Care, King’s College London, London.; NIHR clinician scientist and honorary consultant in palliative medicine, Cicely Saunders Institute for Palliative Care, Policy & Rehabilitation, Florence Nightingale Faculty of Nursing, Midwifery & Palliative Care, King’s College London, London.; NIHR clinician scientist and honorary consultant in palliative medicine, Cicely Saunders Institute for Palliative Care, Policy & Rehabilitation, Florence Nightingale Faculty of Nursing, Midwifery & Palliative Care, King’s College London, London.

**Keywords:** dementia, end of life, family practice, palliative care, primary health care, hospitalisation

## Abstract

**Background:**

Reducing hospital admissions among people dying with dementia is a policy priority.

**Aim:**

To explore associations between primary care contacts, continuity of primary care, identification of palliative care needs, and unplanned hospital admissions among people dying with dementia.

**Design and setting:**

This was a retrospective cohort study using the Clinical Practice Research Datalink linked with hospital records and Office for National Statistics data. Adults (>18 years) who died between 2009 and 2018 with a diagnosis of dementia were included in the study.

**Method:**

The association between GP contacts, Herfindahl–Hirschman Index continuity of care score, palliative care needs identification before the last 90 days of life, and multiple unplanned hospital admissions in the last 90 days was evaluated using random-effects Poisson regression.

**Results:**

In total, 33 714 decedents with dementia were identified: 64.1% (*n* = 21 623) female, mean age 86.6 years (SD 8.1), mean comorbidities 2.2 (SD 1.6). Of these, 1894 (5.6%) had multiple hospital admissions in the last 90 days of life (increase from 4.9%, 95% confidence interval [CI] = 4.2 to 5.6 in 2009 to 7.1%, 95% CI = 5.7 to 8.4 in 2018). Participants with more GP contacts had higher risk of multiple hospital admissions (incidence risk ratio [IRR] 1.08, 95% CI = 1.05 to 1.11). Higher continuity of care scores (IRR 0.79, 95% CI = 0.68 to 0.92) and identification of palliative care needs (IRR 0.66, 95% CI = 0.56 to 0.78) were associated with lower frequency of these admissions.

**Conclusion:**

Multiple hospital admissions among people dying with dementia are increasing. Higher continuity of care and identification of palliative care needs are associated with a lower risk of multiple hospital admissions in this population, and might help prevent these admissions at the end of life.

## INTRODUCTION

Dementia is one of the leading causes of death in high-income nations,^1^ and the number of people dying with dementia requiring symptom management is projected to increase.^2^^,^^3^ It is essential, for patients and the system, to better understand how to provide high-quality end-of-life care for this population.

People with dementia experience a rapid increase in symptoms,^4^^,^^5^ emergency department visits,^6^^,^^7^ and hospital admissions in their last year of life.^8^^,^^9^ Transitions to hospital among people dying with dementia have been associated with markers of poor-quality end-of-life care,^10^ and poor health outcomes such as delirium, falls, and cognitive and functional decline.^11^^–^^13^ Multiple hospital admissions in the last 90 days of life has been suggested as an indicator of poor end-of-life care in people with dementia.^14^^,^^15^

Primary care services, including GPs, nurses, or other healthcare services provided in the community, are likely to contribute to reducing unnecessary transitions at the end of life by providing timely access to patient-centred care.^16^ Community-based palliative care services have been associated with fewer hospital admissions in people with dementia in Australia^17^ and the US.^18^ However, people with dementia experience several barriers to access palliative care services.^19^ Being the first point of contact, GPs play an important role in providing end-of-life care, and contacts with GPs^20^ and home health care^21^ have been associated with lower risk of end-of-life admissions to hospital among older adults and people with dementia. It is not known how this relationship is affected by the frequency and length of contacts,^21^ the level of continuity of care experienced,^22^^,^^23^ or whether palliative care needs are being identified by the GP.^24^

The aim of this study was 1) to describe primary care service use among individuals with dementia in the last year of life, and 2) explore associations between contacts, continuity of care with GPs, palliative care needs identification, and unplanned hospital admissions in people dying with dementia in their last 90 days of life.

## METHOD

### Design and data sources

This is a nationwide population-based retrospective cohort study in England using the Clinical Practice Research Datalink (CPRD), linked with hospital records and mortality data from the Office for National Statistics. CPRD contains anonymised medical records from over 19 million people enrolled in 952 general practices across the UK.^25^ People currently registered in CPRD primary care practices represent approximately 4.6% of the UK population.^26^

**Table:** How this fits in

People with dementia are at high risk of multiple hospital admissions at the end of life and preventing these admissions is a policy priority. This study found that people with dementia who had better continuity of care with GPs were less likely to have multiple hospital admissions in the last 90 days of life, in particular if they lived at home and had multiple comorbidities. People living in care homes and with an identification of palliative care needs in their primary care records were less likely to experience these admissions.

### Population

People included in the study were adults (≥18 years) who died between 1 January 2009 and 31 December 2018, had a dementia diagnosis recorded in primary care or hospital records, and a 12-month before death registration period in a GP practice with continuous high-quality data based on CPRD quality checks.^25^ Dementia diagnosis was identified from primary care records (using Read codes, standard clinical codes used in UK GP practices to record diagnosis and procedures)^27^ and hospital records (using International Statistical Classification of Diseases and Related Health Problems 10 [ICD-10] codes), based on previous studies^28^ (see Supplementary Box S1 and Table S1).

### Outcome

The primary outcome was multiple unplanned admissions to hospital in the last 90 days of life (0 if no, 1 if yes), based on Gozalo *et al* as either more than two unplanned admissions for any reason or more than one unplanned admission for respiratory infection, urinary tract infection, dehydration, or sepsis^10^ (see Supplementary Box S2).

### Explanatory variables

To describe primary care service use, the number of participant’s consultations with a GP in the last 12 months of life were determined. Face-to-face and telephone consultations were included regardless of where the consultation took place (practice, home, or out-of-hours).

An exposure period was defined between months 12 and 4 before death (day 365 until day 91 before death). To account for the fact that participants in hospital cannot visit their GP, a rate of consultations with GPs by month was calculated by dividing the total number of consultations with GPs by the number of days participants were in the community (excluding days in hospital) during the exposure period (Box 1).

**Box 1.** Formulas for calculating rate GP contacts and continuity of care score

$\begin{array}{l} \text{Rate}\;\text{GP}\;\text{contacts}\;\text{per}\;\text{month}:\\ =\frac{\text{number}\;\text{of}\;\text{contacts}\;\text{with}\;\text{a}\;\text{GP}\;\text{between}\;\text{months}\;12\;\text{to}\;4\;\text{before}\;\text{death}}{\left(271-\text{number}\;\text{days}\;\text{in}\;\text{hospital}\;\text{between}\;\text{months}\;12\;\text{to}\;4\;\text{before}\;\text{death}\right)}\times 30 \end{array}$ $\begin{array}{l} \mathrm{Continuity}\;\mathrm{of}\;\mathrm{care}(\mathrm{CoC})\mathrm{score}:\\ \mathrm{Coc}=\frac{\left(\sum n_{i}^{2}-N\right)}{N(N-1)} \end{array}$*n_i_* = the number of contacts the patient had with a GP during the exposure period (months 12 and 4 before death)*N* = the total number of GP contacts during exposure period.

The Consultation and Staff files provided by CPRD include an anonymised code identifying the physician who recorded each consultation, which was used to calculate the Herfindahl–Hirschman Index continuity of care score (Box 1).^29^ This continuity of care score measures the extent to which consultations during a certain period of time are with the same physician, and has a range from 0 to 1 (1 means all contacts the patient has in that period were with the same GP). Only contacts with GPs during the exposure period were considered.

Identification of palliative care needs was derived from the Palliative Care Register, an electronic register introduced in 2004 in England that aims to identify people in the GP practice who might benefit from a palliative care approach.^30^ When patients are identified as having palliative care needs, their GP adds a code in the patients’ clinical records that is then captured by the Palliative Care Register. In this study, people with a relevant code at any point before the last 90 days of life were identified in order to recognise people who had been identified by their GPs as having palliative care needs (see Supplementary Table S2).^27^^,^^31^^–^^33^

### Covariables

Factors associated with multiple hospital admissions in the last 90 days of life were examined based on previous research and theoretical models.^34^^,^^35^ Age at death was calculated using the year of death and year of birth. Sex and GP practice region were extracted from the CPRD. The 2011 England and Wales rural–urban classification of the GP practice where participants were enrolled and the 2015 English Index of Multiple Deprivation quintiles at lower super output areas level from the latest available postcode of residence for participants were used.

The underlying cause of death, place, and date of death were identified from the Office for National Statistics. The underlying cause of death was grouped into ICD-10 block codes (see Supplementary Table S3). The number of comorbidities (excluding dementia) were calculated using the count of chronic diseases from the Quality and Outcomes Framework Read codes rules (see Supplementary Table S2).^27^^,^^36^ Read codes were used to identify whether participants had a record of living in a care home (nursing or residential care home) based on previous publications (see Supplementary Table S4).^37^

### Analysis

Changes in the annual proportion of participants with multiple hospital admissions in the last 90 days of life between 2009 and 2018 were explored using a scatter plot, with the proportion of multiple hospital admissions by year of death adjusted by age and sex. The mean (95% confidence interval) number of contacts with GPs by month before death was explored.

A multilevel Poisson regression with robust error variance and a random intercept for the region and participant’s GP practice was used to estimate the association between the rate of GP contacts per month, continuity of care score, identification of palliative care needs during the exposure period, and multiple hospital admissions in the last 90 days of life. As the Herfindahl–Hirschman continuity of care score can only be calculated with at least two contacts, participants with fewer than two contacts with GPs were excluded (2920/33 714, 8.7%). Missing values for covariables were small (<1%) and therefore excluded.

A subgroup analysis was performed to explore the influence of sociodemographic and illness-related factors on the association between the rate of GP contacts per month, continuity of care, identification of palliative care needs, and the outcome.

Three sensitivity analyses were conducted:

- as the continuity of care score has been shown to be less stable when participants have <4 contacts, an analysis excluding those participants was performed;
- to explore the effect of excluding participants with <2 contacts with GPs, the same general multivariate model was performed excluding continuity of care; and
- an analysis was performed including people with at least 1 day of enrolment during the last year of life (*n* = 57 659). As a notable proportion of people with <365 days of enrolment had <2 contacts with GPs, the continuity of care score was excluded in this analysis.

All analysis were performed using Stata® version 16.1.

## RESULTS

### Characteristics of the study sample

This study identified 57 659 people with dementia who died between 2009 and 2018, and who were registered in a GP practice during the last year of life. After excluding 23 945 people without a complete year of registration before death, 33 714 participants were included in the analysis (Supplementary Figure S1).

Demographic characteristics for people with and without a complete year of registration are described in Supplementary Table S5. The cohort had an average age at death of 86.6 years (SD 8.1), 64.1% (*n* = 21 623) were female, 21.5% (*n* = 7260) lived in the least deprived quintile, and 56.0% (*n* = 18 896) of the cohort had a code for living in a care home. The most common underlying cause of death was dementia (36.8%, *n* = 12 404) followed by cerebrovascular disease (10.7%, *n* = 3615), and cancer (8.7%, *n* = 2926) (Table 1).

**Table 1.** Characteristics of participants by multiple hospital admissions in the last 90 days

	**Total**	**Multiple unplanned hospital admissions last 90 days**	

		**No**	**Yes**
**Total, *n***	33 714	31 820 (94.4)	1894 (5.6)

**Age, mean (SD)**	86.56 (8.07)	86.69 (8.01)	84.67 (8.79)

**Sex, *n* (%)**			
Male	12 091 (35.9)	11 171 (35.1)	920 (48.6)
Female	21 623 (64.1)	20 649 (64.9)	974 (51.4)

**IMD quintiles, *n* (%)**			
(Least deprived) 1	7260 (21.5)	6906 (21.7)	354 (18.7)
2	7451 (22.1)	7062 (22.2)	389 (20.5)
3	7837 (23.2)	7459 (23.4)	378 (20.0)
4	5895 (17.5)	5549 (17.4)	346 (18.3)
5	5258 (15.6)	4831 (15.2)	427 (22.5)
Missing	13	13	0

**Lived in care home, *n* (%)**			
No	14 818 (44.0)	13 816 (43.4)	1002 (52.9)
Yes	18 896 (56.0)	18 004 (56.6)	892 (47.1)

**Rurality, *n* (%)**			
Urban	28 921 (85.8)	27 192 (85.5)	1729 (91.3)
Rural	4793 (14.2)	4628 (14.5)	165 (8.7)

**Region, *n* (%)**			
North East	703 (2.1)	666 (2.1)	37 (2.0)
North West	5947 (17.6)	5510 (17.3)	437 (23.1)
Yorkshire & The Humber	1047 (3.1)	981 (3.1)	66 (3.5)
East Midlands	364 (1.1)	342 (1.1)	22 (1.2)
West Midlands	4028 (11.9)	3785 (11.9)	243 (12.8)
East of England	2755 (8.2)	2618 (8.2)	137 (7.2)
South West	4855 (14.4)	4678 (14.7)	177 (9.3)
South Central	5311 (15.8)	5121 (16.1)	190 (10.0)
London	3386 (10.0)	3107 (9.8)	279 (14.7)
South East Coast	5318 (15.8)	5012 (15.8)	306 (16.2)

**Cause of death, *n* (%)**			
Dementia	12 404 (36.8)	11 917 (37.5)	487 (25.7)
Cancer	2926 (8.7)	2731 (8.6)	195 (10.3)
Cerebrovascular disease	3615 (10.7)	3429 (10.8)	186 (9.8)
Ischaemic heart disease	2510 (7.4)	2363 (7.4)	147 (7.8)
Influenza and pneumonia	1800 (5.3)	1657 (5.2)	143 (7.6)
Chronic pulmonary disease	1148 (3.4)	1003 (3.2)	145 (7.7)
Chronic heart disease	1160 (3.4)	1087 (3.4)	73 (3.9)
Parkinson’s disease	831 (2.5)	801 (2.5)	30 (1.6)
Senility	750 (2.2)	737 (2.3)	13 (0.7)
Other	6565 (19.5)	6090 (19.1)	475 (25.1)
Missing	5	5	0

**Number of QoF comorbidities, mean (SD)**	2.23 (1.60)	2.20 (1.59)	2.72 (1.69)

**Place of death, *n* (%)**			
No home	30 376 (90.1)	28 577 (89.8)	1799 (95.0)
Home	3338 (9.9)	3243 (10.2)	95 (5.0)

*IMD = Index of Multiple Deprivation. QoF = Quality and Outcomes Framework. SD = standard deviation.*

**Figure 1.**
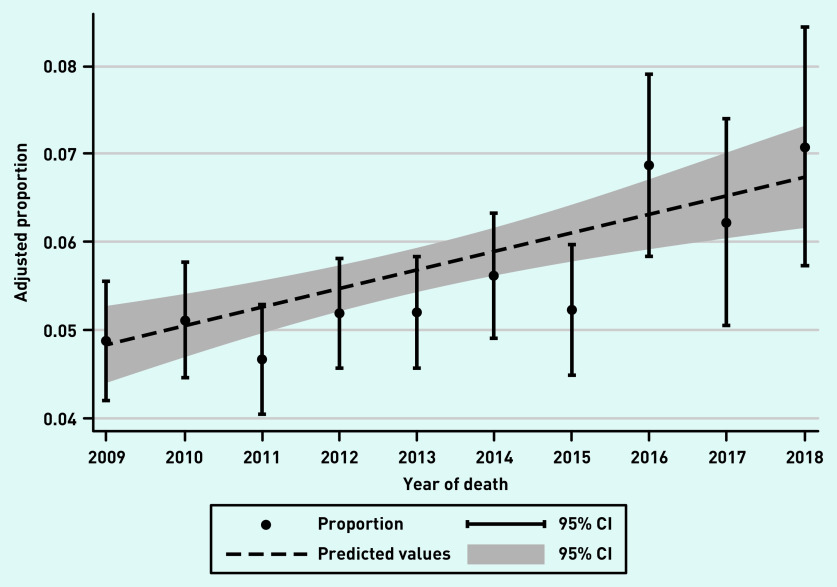
*Age-and sex-adjusted proportion of decedents who experienced multiple hospital admissions in the last 90 days of life by year of death. CI = confidence interval.*

Of 33 714 participants, 1894 (5.6%) had multiple hospital admissions in the last 90 days of life. This proportion increased from 4.9% (95% CI = 4.2 to 5.6) in 2009 to 7.1% (95% CI = 5.7 to 8.4) in 2018 (Figure 1). The mean continuity of care score in the cohort was 0.41 (SD 0.30). Participants with multiple hospital admissions in the last 90 days had lower continuity of care scores than those without these admissions (Table 2). There were 3169 (9.4%) participants who were identified as having palliative care needs by a GP before their last 90 days of life, and they were less likely to have multiple hospital admissions in the last 90 days (Table 2).

**Table 2.** Association between GP contacts, continuity of care score, identification of palliative care needs, and multiple hospital admissions

	**Multiple hospital admissions in the last 90 days**								

	**No (*n* = 31 820)**			**Yes (*n* = 1894)**			**IRR^a^**	**95% CI**	***P*-value**

	**Events, *n***	**Days in community, *n***	**IR × 30 days**	**Events, *n***	**Days in community, *n***	**IR × 30 days**			
**GP contact rate (12–4 months before death)**	313 501	8 395 512	1.12	21 259	488 090	1.31	1.08	(1.05 to 1.11)	<0.001

	**Mean**	**SD**	**IMD**	**Mean**	**SD**				

**Continuity of care score (12–4 months before death)**	0.41	0.30		0.38	0.28		0.79	(0.68 to 0.92)	0.003

	**Freq**	**%**		**Freq**	**%**				

**Palliative care QoF any time before last 90 days**									
No	28 782	90.5		1763	93.1				
Yes	3038	9.5		131	6.9		0.66	(0.56 to 0.78)	0.001

a *Multilevel Poisson model with a random intercept for region and GP practice, adjusted by age, number of QoF comorbidities, sex, IMD, rurality, living in a care home, cause of death, and year of death. The model includes only participants with at least two contacts with the GP during the exposure period. The full model is available in Supplementary Table S7. CI = confidence interval. IR = incidence rate. IRR = incidence risk ratio. IMD = Index of Multiple Deprivation. QoF = Quality and Outcomes Framework. SD = standard deviation.*

**Figure 2.**
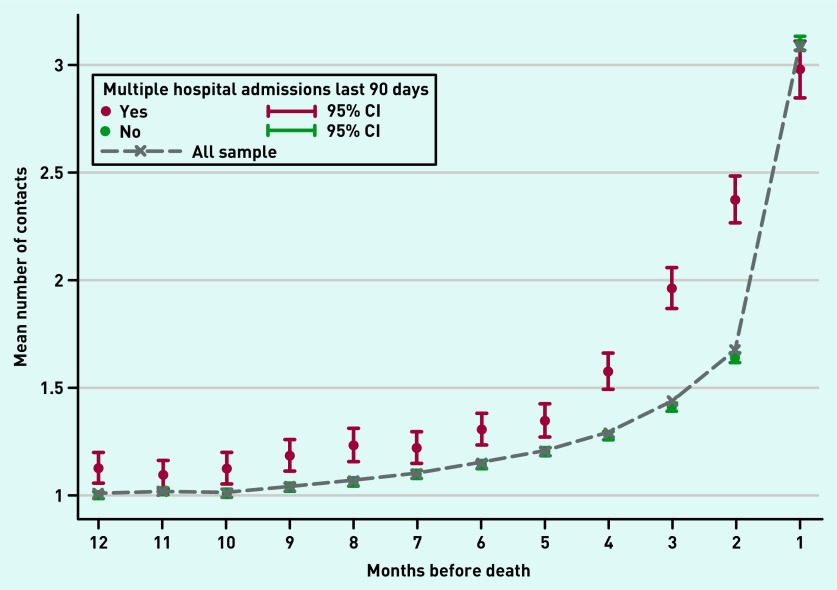
*GP contacts for participants with and without multiple hospital admissions by month before death. Mean number of contacts with GPs in the last 12 months of life for participants with dementia with and without multiple hospital admissions in the last 90 days by month before death. CI = confidence interval.*

Participants had on average 16.1 (SD 11.6) contacts with GPs in their last year of life, which increased closer to death, particularly in the last month of life (mean 3.1, SD 2.9). Participants with multiple hospital admissions had a higher mean number of contacts with GPs throughout the whole last year of life, except for the last month before death (Figure 2).

### Multilevel adjusted model

In the adjusted model, participants with a higher rate of contacts with GPs per month were more likely to have multiple hospital admissions in the last 90 days of life. Participants with greater continuity of care scores and identification of palliative care needs were less likely to have multiple hospital admissions (Table 2).

**Figure 3.**
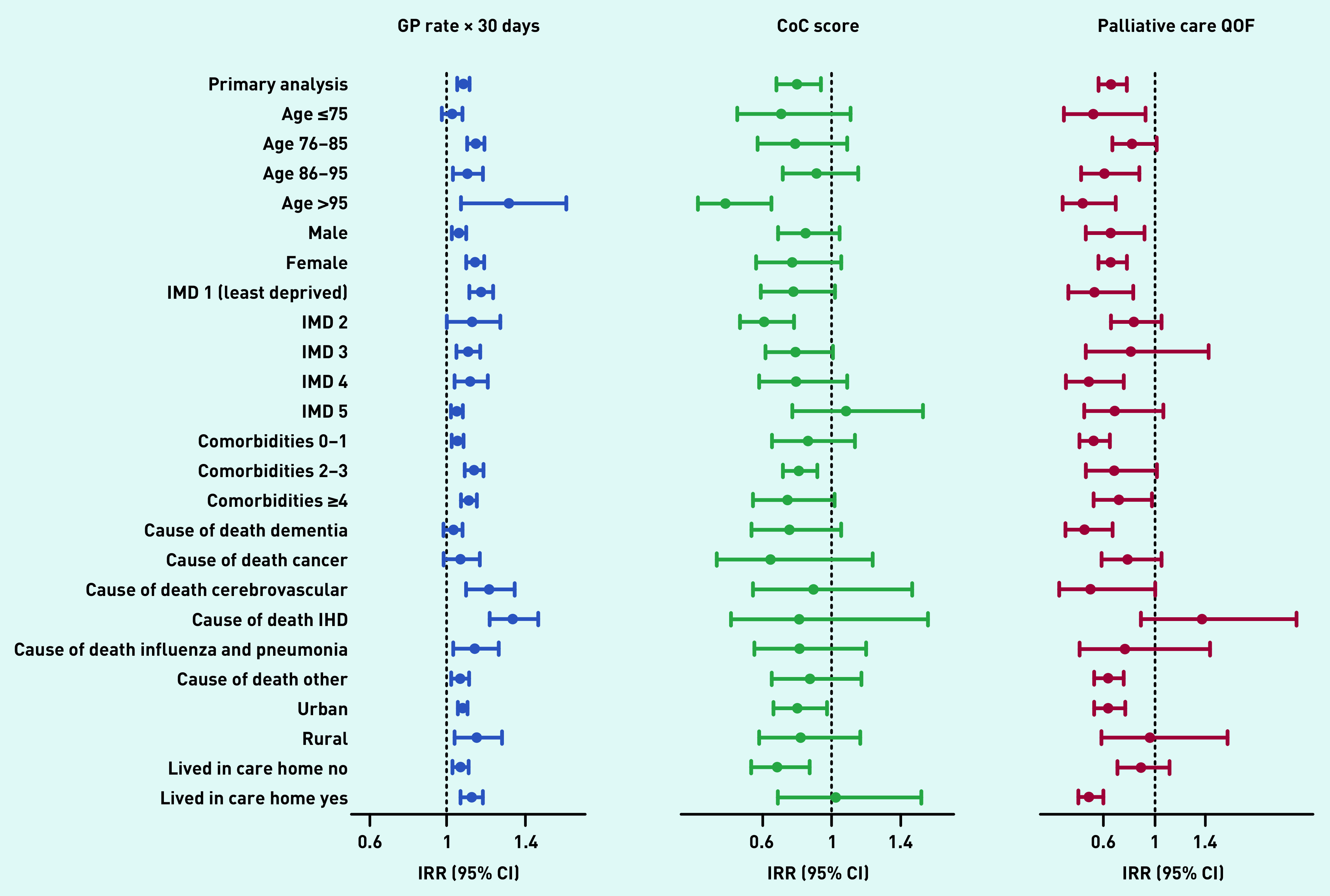
*Subgroup analysis. The figure shows results from the subgroup analyses exploring the influence of sociodemographic and illness-related factors in the association between the rate of GP contacts per month, continuity of care score, identification of palliative care needs before the last 90 days of life, and multiple hospital admissions in the last 90 days of life (primary analysis). The IRR represents the risk of multiple hospital admissions for the monthly rate of GP contacts, level of continuity of care, and identification of palliative care needs before the last 90 days of life. All models are adjusted for age in years, number of comorbidities, IMD quintile, underlying cause of death, rurality, sex, living in care homes, and year of death, excluding the variable used for the subgroup analysis, and include a random intercept for region and GP practice. CI = confidence interval. CoC = continuity of care. IHD = ischaemic heart disease. IMD = Index of Multiple Deprivation. IRR = incidence risk ratio. QoF = Quality and Outcomes Framework.*

The subgroup analysis showed the positive association between the number of contacts with GPs and multiple hospital admissions in the last 90 days was significant for all groups except for participants <75 years of age, and for those whose underlying cause of death was dementia or cancer (Figure 3 and Supplementary Table S6). Better continuity of care with GPs was associated with a lower risk of multiple hospital admissions mainly for participants >95 years, with more comorbidities, living in urban areas, and not living in care homes. Identification of palliative care needs was associated with a lower risk of multiple admissions in older participants (>85 years old), those with no comorbidities, living in urban areas and in care homes, and for those whose underlying cause of death was dementia (Figure 3 and Supplementary Table S6).

All sensitivity analysis performed showed similar results (see Supplementary Table S7).

## DISCUSSION

### Summary

In this large population-based cohort of people who died with dementia in England, results show that more contacts with GPs was positively associated with multiple hospital admissions in the last 90 days of life, whereas continuity of care with GPs and identification of palliative care needs were negatively associated with these hospital admissions. Continuity of care was particularly relevant for participants >95 years of age, those with more comorbidities, living in urban areas, and not living in care homes. Identification of palliative care needs was particularly relevant in participants without comorbidities, and those living in urban areas and care homes.

### Strengths and limitations

This study uses a large nationwide population-based cohort linked with hospital and death certificates records. Participants with dementia were identified from primary and hospital care records, reducing the risk of missing people with incomplete records.

This study has some limitations. Restricting the sample to participants with a full year of enrolment in a GP practice is likely to exclude people who changed their GP practice because of deterioration or severe cognitive impairment.^38^^,^^39^ However, the sensitivity analysis including people with <365 days of enrolment showed similar results. Information on the appropriateness of admissions to hospital, quality of care, or reasons for GP visits, which are likely to influence the risk of end-of-life admissions, was not available. Palliative Care Quality and Outcomes Framework codes were used to identify people who have been recognised as having palliative care needs in primary care. However, these codes do not identify all people whose death is anticipated by the GP.^33^

The measure of continuity of care used in this study does not capture the nature of the relationship between physicians and patients or the quality of care received.^40^^,^^41^ Although other measures of continuity of care exist, the Herfindahl–Hirschman Index continuity of care score has been widely used in the literature and does not rely on the need to identify a usual provider.^29^^,^^41^^–^^43^

### Comparison with existing literature

Studies investigating the association between contacts with GPs and hospital use at the end of life show conflicting results. Contacts with GPs have been positively associated with end-of-life hospital admissions among people with cancer in Canada^22^ and older adults in Australia,^44^ and negatively associated with end-of-life hospital admissions in the US in patients with congestive heart failure and chronic obstructive pulmonary disease.^20^ In Chen *et al*,^21^ people with dementia receiving home health care in Taiwan had a higher risk of multiple hospital admissions in the last 90 days. However, this effect varied depending on the frequency and duration of home health care. Differences in results could also be explained by differences in healthcare systems^45^ and type of conditions. People with frequent hospital admissions are likely to have more GP contacts because of higher healthcare needs. However, it is possible that more GP contacts might reflect poor coordination and integration between healthcare services. More research is needed to understand how interdisciplinary work between GPs and other community care services might have an impact on admissions to hospital in this population.^46^^,^^47^

Two studies (both on cancer) have explored the relationship between continuity of care and hospital admissions for people approaching the end of life in Canada^22^ and England^48^ with similar findings to the current study. Continuity of care can increase trust between patients and doctors, increase adherence to long-term treatments, improve the quality of management, and reduce over-aggressive treatment, in particular among people with multimorbidity and those living in the community.^49^^–^^52^ Continuity of care was low in the sample of this study (mean continuity of care score 0.4). This is similar to findings from a study in older adults in the UK.^53^ Indices that measure concentration of care, such as the continuity of care score used in this study, are highly influenced by the number of contacts and type of professional considered (GP versus specialists), which could explain differences across studies.

### Implications for research and practice

Despite the evidence for the potential benefit of continuity of care,^54^ the proportion of patients who were able to see their preferred GP has declined by 9% between 2012 and 2017 in England.^55^ Strategies such as assigning a key worker,^34^ assigning patients to small multidisciplinary teams within a practice, enhancing the role of receptionists to support continuity, and prioritising continuity for patients who may benefit the most have been recommended to improve the level of continuity of care,^56^ and might help prevent unnecessary admissions to hospital in older people with dementia and multimorbidity living at home.

The finding of this study suggests that identifying people who might benefit from a palliative care approach could help to reduce unnecessary end-of-life transitions to hospital. These findings are consistent with results from a previous study in a London population.^24^ However, recognising when people with dementia are approaching the end of life is challenging, and GPs have reported barriers to doing so, such as lack of knowledge and training.^57^^,^^58^ Screening tools such as the Supportive and Palliative Care Indicators Tool or the Electronic Frailty Index developed in the UK for primary care settings,^59^^,^^60^ and the IPOS-Dem developed specially for people with dementia,^61^^,^^62^ might help GPs flag people with high risk of deteriorating and dying, assess patients’ needs, and identify those who might benefit from a palliative care approach.

## Data

The data that support the findings of this study are available from the CPRD. Access to CPRD data is subject to protocol approval by an Independent Scientific Advisory Committee (ISAC). The protocol of this study was approved by the ISAC in April 2021 (Protocol 20_031).
